# Significance of Neonatal Heart Rate in the Delivery Room—A Review

**DOI:** 10.3390/children10091551

**Published:** 2023-09-14

**Authors:** Ellisiv Nerdrum Aagaard, Anne Lee Solevåg, Ola Didrik Saugstad

**Affiliations:** 1 Division of Pediatric and Adolescent Medicine, Oslo University Hospital Rikshospitalet, 0424 Oslo, Norway; ellaag@ous-hf.no (E.N.A.); a.l.solevag@medisin.uio.no (A.L.S.); 2 Department of Pediatric Research, University of Oslo, 0424 Oslo, Norway; 3 Department of Pediatrics, Robert H Lurie Medical Research Center, Northwestern University, Chicago, IL 60611, USA

**Keywords:** infants, newborn, neonatal resuscitation, heart rate, pulse oximetry, electrocardiogram

## Abstract

Background: Heart rate (HR) is considered the main vital sign in newborns during perinatal transition, with a threshold of 100 beats per minute (bpm), below which, intervention is recommended. However, recent changes in delivery room management, including delayed cord clamping, are likely to have influenced normal HR transition. Objective: To summarize the updated knowledge about the factors, including measurement methods, that influence HR in newborn infants immediately after birth. Additionally, this paper provides an overview of delivery room HR as a prognostic indicator in different subgroups of newborns. Methods: We searched PubMed, EMBASE, and Google Scholar with the terms infant, heart rate, delivery room, resuscitation, pulse oximetry, and electrocardiogram. Results: Seven studies that described HR values in newborn infants immediately after birth were included. Pulse oximetry-derived HR percentiles after immediate cord clamping may not be applicable to the current practice of delayed cord clamping and the increasing use of delivery room electrocardiograms. Mask ventilation may adversely affect HR, particularly in premature and non-asphyxiated infants. Prolonged bradycardia is a negative prognostic factor, especially if combined with hypoxemia in infants <32 weeks of gestation. Conclusions: HR assessment in the delivery room remains important. However, the cardiopulmonary transition is affected by delayed cord clamping, gestational age, and underlying conditions.

## 1. Introduction

Heart rate (HR) is considered the gold standard clinical indicator of successful transition from intra- to extrauterine life and is used to guide delivery room resuscitation and stabilization [1]. International guidelines recommend an HR threshold of 100 beats per minute (bpm), below which, intervention should be considered. Current guidelines recommend neonatal HR assessment by cardiac auscultation, pulse oximetry, and/or electrocardiogram (ECG) [1]. However, studies indicate that some methods, including auscultation and palpation [2,3,4], may be inaccurate. This is important, as HR overestimation might result in interventions being delayed, while underestimation might result in unwarranted interventions [2]. Also, with contemporary methods for fast and reliable measurement, the HR threshold for intervention in different subgroups of infants, e.g., by the cord management method [5,6], delivery mode [7], and gestational age [7], has been challenged. As HR is dependent on blood oxygenation, changes in practice with regard to the use of supplementary oxygen in preterm and term infants may have affected the expected course of and prognostic value of HR in the first minutes after birth. A pulse oximeter continuously measures oxygen saturation and HR; the signal is delayed compared to dry-electrode ECG/newer generation ECG devices. The aim of this review was to summarize the updated knowledge about factors that influence HR, including measurement methods, in newborn infants. Additionally, it provides an overview of recent studies about delivery room HR as a prognostic indicator in different subgroups of newborns.

## 2. Methods

We searched for articles in PubMed, EMBASE, and Google Scholar until July 2023 using search terms including “infant”, “heart rate”, “delivery room”, “resuscitation”, “pulse oximetry”, and “electrocardiography”. Additional articles were identified via screening of the reference lists of identified articles. Articles were evaluated based on title, abstract, and methods section and included if they addressed HR in the first 5–10 min of life. Human studies in all languages were assessed.

## 3. Results

Seven studies that described HR values in mainly non-ventilated newborn infants immediately after birth were included [5,8,9,10,11,12,13]. Characteristics of these studies are presented in Table 1. HR values for the first 5 min (300 s) of life from the same seven studies are presented in Table 2. We included additional studies about prognosis related to HR in different subgroups of newborns.

### 3.1. Factors, Including Measurement Method, That Influence HR in Newborn Infants Immediately after Birth

#### 3.1.1. First Reports of Normal HR Immediately after Birth—Pulse Oximetry

During neonatal resuscitation, the use of pulse oximetry to obtain preductal oxygen saturation and HR is recommended [1]. In the 1980s, several studies published oxygen saturation data in the first few minutes after birth [14]. Dawson et al. reported HR [9] in addition to oxygen saturation [7] percentiles in the first 10 min of life in healthy term and preterm infants. These studies demonstrated that HR starts out low and increases rapidly in the first minutes of life. There is a wide normal range and a slower HR increase in preterm vs. term infants, after cesarean vs. vaginal birth, and after maternal analgesia administration [9]. According to these data, the median (interquartile range, IQR) HR for term infants at one minute of age is 99 (66–132) bpm versus 96 (72–122) bpm in preterm infants (Table 2). A plateau of 160 (range 130–180) bpm was, in most cases, reached before 10 min of age in healthy term babies. At one minute of age, 61% of healthy term infants had an HR < 100 bpm, decreasing to 21% and 7% at 2 and 3 min of age, respectively. However, still, at 10 min of age, 1% of healthy term infants had an HR < 100 bpm [9]. Singh et al. [15] and Kamlin et al. [16] investigated the accuracy of the pulse oximetry HR measurement in preterm infants (*n* = 30) in the Neonatal Intensive Care Unit (NICU) and late preterm and term infants (*n* = 55) in the delivery room, respectively. Both Singh et al. [15] and Kamlin et al. [16] found lower HRs with pulse oximetry compared to ECGs. On the other hand, pulse oximetry detected an HR < 100 bpm only 89% of the time [16].

#### 3.1.2. Recent Reports of Normal HR Immediately after Birth—Electrocardiogram

With traditional ECGs, self-adhesive electrodes on clean and dry skin are attached to a monitor that measures and displays the heart’s electrical activity. Two papers reported the time to a reliable HR display, comparing pulse oximetry with ECGs during the resuscitation of preterm (*n* = 46) and term (*n* = 20) infants [17,18]. Both studies concluded that the time to obtain an ECG-derived HR was half that of pulse oximetry (median time 28 s (ECG) vs. 62 s (pulse oximetry) [17]). Similarly, van Vonderen et al. [12] showed that the mean (standard deviation, SD) time from birth until an HR was detected using an ECG and pulse oximetry was 82 (26) s and 99 (33) s, respectively, in 53 vaginally- and caesarian section-delivered preterm and term infants. Bradycardia was diagnosed twice as often in the first two minutes of life via pulse oximetry vs. ECGs with a mean difference in HR between pulse oximetry and ECGs of −3 bpm in the first 10 min of life.

Kukka et al. [13] used a dry-electrode ECG, a technology not contingent on skin cleaning and drying prior to application, and made HR percentile charts based on 1155 crying and 54 non-crying but breathing infants ≥ 33 weeks’ gestation (Table 1 and Table 2). Similarly, Bjorland et al. [11] used the same technology to describe HR in the first 5 min of life in 898 vaginally delivered term infants. Both studies demonstrated that HR could be detected earlier (at 10 s and 5 s of life, respectively) than with traditional ECGs. The HR was consistently higher than the pulse oximetry-derived references by Dawson et al. [9] (Table 2).

Thus, studies indicate that an ECG provides an HR faster than pulse oximetry. However, potential limitations of ECGs include that pulseless electric activity may be misinterpreted as an HR and thus might delay resuscitative interventions [17,19,20]. The fact that recent studies using dry-electrode ECGs show an HR difference (Table 2) compared to pulse oximetry by far exceeding the 3 bpm found in studies with paired measurements [12] should be further explored.

#### 3.1.3. Delayed Cord Clamping

While traditionally, HR has been seen as a pure marker of fetal and neonatal hypoxia, there is an increasing awareness that other factors, including cardiac preload, may play an important role [21]. When the umbilical cord is clamped, the infant is separated from the low-resistance placental circulation, systemic vascular resistance and blood pressure increase, and right-to-left ductal shunting decreases [22]. Lung aeration after birth results in reduced pulmonary vascular resistance and increased pulmonary blood flow. Thus, lung aeration prior to umbilical cord clamping ensures that the left ventricular preload and output are maintained when the umbilical cord is clamped [23].

Smit et al. [5] reported on the pulse oximetry-derived HR during 109 midwife-attended home deliveries and concluded that delayed cord clamping and/or skin-to-skin contact were associated with lower HRs and a slower HR increase compared to the reference ranges by Dawson et al. [9] (Table 2). Pichler et al. [6] confirmed that the pulse oximetry-derived HR was lower in caesarian section-delivered preterm infants with delayed (for 30 s or 60 s) cord clamping vs. immediate cord clamping. In a study from Nepal by Kc et al. [24], the HR was 9 and 3 beats lower at 1 and 5 min, respectively, in late preterm and term infants after delayed vs. immediate cord clamping. Physiologically based cord clamping, i.e., cord clamping after established lung aeration, did not result in less bradycardia in infants ≥32 weeks needing resuscitation compared to early cord clamping [10].

#### 3.1.4. The Trigemino-Cardiac Reflex

PPV with a facemask may cause a powerful vagal stimulus and result in reflex bradycardia [25]. In delivery room resuscitation, placing a facemask over the mouth and nose may activate the trigeminal nerve and stimulate the trigemino-cardiac reflex, which is characterized by blood pressure changes, bradycardia, and apnea [26]. In support of this, a cohort study showed that 54% of preterm infants had less spontaneous breathing after facemask placement [26]. In infants >34 weeks’ gestation, Gaertner et al. [27] reported in a subgroup analysis of a previously conducted clinical trial that 10% of delivery room facemask applications were followed by apnea and bradycardia, suggesting that the trigemino-cardiac reflex is less pronounced in late preterm and term infants.

#### 3.1.5. Heart Rate in Hypoxemia and Asphyxia

In asphyxiated infants, HR, in addition to oxygen saturation, Apgar scores, and time to first breath, has been shown to be different in infants with a poor prognosis [28], and an HR < 100 bpm is a major criterium for starting PPV immediately after birth. However, according to the Dawson references [9] (Table 2), it is quite clear that not all newborn infants with an HR < 100 bpm have suffered birth asphyxia and are in need of PPV. In addition to HR itself, it is therefore important to make a clinical assessment of the infant and assess the ventilatory drive and how vigorous it is before PPV is initiated.

In the classic resuscitation/oxygen studies in the 1990s and early 2000s, HR (auscultation) was in general <100 bpm in newly born infants given PPV. The Resair 2 study [29] included 609 term or near-term newly born infants in need of PPV and pseudorandomized to receive 100% or 21% oxygen. The major inclusion criterion was an HR < 80 bpm. At one minute of age, 2/3 had an HR < 100 bpm, with a median of 90 (95% CI 40–140) bpm. From 3 min and onward, the HR was stable above 130 bpm [28]. Ramji et al. [30] found a mean (SD) HR of 94 (26) and 87 (28) bpm at one minute of age in infants in need of PPV given air and 100% oxygen, respectively [31]. In a study from Uganda, Pejovic et al. [32] found a mean HR of 82 at 30 s of life and 100 at 60 s of life for newly born infants needing bag and mask ventilation immediately after birth. Sixty-three percent had an HR < 100 bpm at 1 min of age [32].

In a study of 98 infants ≥30 weeks’ gestation with inadequate respiration and therefore needing PPV at birth, Kibsgaard et al. [33] found that in ventilated infants, the median (IQR) first measured HR was 112 (80, 146) bpm recorded 30 (15, 52) s after birth by a dry-electrode ECG. The same group of investigators found an HR > 100 bpm in about 60% of the infants receiving PPV [34]. They concluded that an HR ≥ 100 bpm does not necessarily imply that the newborn is not in need of resuscitation.

Several studies have demonstrated no significant difference regarding the development of HR in the first minutes after birth both in term and preterm infants as related to initial FiO_2_ [29,35]. The interaction between HR and hypoxemia and its effect on tissue oxygen delivery and oxygenation immediately after birth has, however, been insufficiently studied. A more thorough understanding of such a relationship might explain the interaction between hypoxemia and bradycardia in the course of perinatal transition. In preterm infants, Bresesti et al. [36] showed that the degree of bradycardia impacted oxygen saturation. Prolonged bradycardia (≥2 min) combined with an oxygen saturation <80% at 5 min of age was associated with a lower oxygen saturation, higher cerebral fractional tissue oxygen extraction, and higher FiO_2_ in the first minutes of life. I.e., only in the case of bradycardia and hypoxemia did cerebral oxygenation drop. Infants with prolonged bradycardia had significantly lower oxygen saturation until 5 min compared to infants with no bradycardia and until 4 min compared to infants with brief bradycardia. After 5 min, there were no differences between the groups [36].

Badurdeen et al. [37] investigated the trigemino-cardiac reflex in late preterm and term infants and found that in initially depressed infants, the application of a facemask resulted in an increased not a decreased HR. These findings prompted the authors to speculate that the trigemino-cardiac reflex is suppressed in asphyxiated infants, i.e., those with a low or unstable baseline HR. In contrast, the infants that experienced an HR decrease upon facemask application had a higher baseline HR.

### 3.2. Delivery Room HR as a Prognostic Indicator in Different Subgroups of Newborns

#### 3.2.1. The Golden Minute

McCarthy et al. [38] found a median (IQR) time to auscultation of the heart of 62 (40–79) s and that the first HR was available after 70 (57–89) s, whereas van Vonderen et al. [12] found that HR was detected at a mean (SD) time of 82 (26) s (ECG) and 99 (33) s (pulse oximetry) after birth. Thus, the principle of the golden minute, implying that HR assessment should be made within 60 s of birth, may not always be feasible with traditional methods of HR assessment. According to guidelines [39], HR assessment should be performed without delay so that bradycardia can be diagnosed, and respiratory support be initiated within 60 s after birth. However, even with the increasing use of ECGs, which have been shown to provide an HR faster than pulse oximetry [33], studies indicate that respiratory support is being initiated later. Badurdeen et al. [37] showed that in late preterm and term infants with an anticipated need for resuscitation, respiratory support was initiated at a median (IQR) age of 63 (41–112) s. As there was a wide range of HRs when respiratory support was initiated, the authors speculated that insufficient respiratory efforts, rather than bradycardia, were common reasons for commencing positive pressure ventilation (PPV), which is in agreement with Kibsgaard et al. [33].

#### 3.2.2. Prognostic Value and Significance of Delivery Room HR Assessment

Yam et al. [40] reported the HR rise during mask ventilation in infants with GA < 30 weeks (*n* = 27). They found a median time of 73 s and 243 s for the HR to reach >100 bpm and >120 bpm, respectively. Similarly, Palme-Kilander and Tunell [41] documented a rapid HR increase after the establishment of the gas exchange. Saugstad et al. [28] reported that a five-minute HR ≤ 60 bpm had an odds ratio (OR) of 16.5 (3.1–86.6) for dying, and those who had resuscitation failure defined as an HR < 100 bpm and/or cyanosis at 90 s of life had a 30% risk of dying the first week of life compared to 7.7% in those without resuscitation failure. Further, survivors of perinatal asphyxia had a significantly higher HR during the first 30 min of life compared to those who did not survive [28]. The initial HR in resuscitated infants >1000 g may therefore be a determinant of early neonatal death and moderate-to-severe brain damage in survivors [28]. Kapadia et al. [42] could demonstrate that in preterm infants <32 weeks GA, mortality increased in a linear fashion with the duration of bradycardia during the first 5 min of life. These authors [42] performed an individual patient data meta-analysis of eight studies that used pulse oximetry in preterm infants with GA < 32 weeks. Newborns with prolonged bradycardia (HR < 100 bpm for ≥2 min) had higher odds of hospital death and also when adjusting for potential confounders. Neonates with an additional oxygen saturation <80% at 5 min of life had higher odds of hospital death: OR 18.6 (4.3–79.7) [42]. Infants who were bradycardic in the delivery room were more premature and had lower birth weights compared to infants without bradycardia. There was no association between initial FiO_2_ and bradycardia. More infants with delivery room bradycardia had an oxygen saturation <80% at 5 min after birth and low 1 min and 5 min Apgar scores. Also, as the bradycardia duration increased, the incidence of intraventricular hemorrhage, bronchopulmonary dysplasia, and in-hospital mortality increased. In another study, infants who did not reach an HR of 100 bpm by 5 min of life were at an increased risk of death (OR 4.57, 95% CI 1.62–13.98, *p* < 0.05) [43].

## 4. Discussion

International resuscitation guidelines recommend repeated assessment of HR during neonatal resuscitation [1]. If the HR is <100 bpm and/or the infant is apneic or breathing irregularly, PPV via a facemask should be commenced. If the HR remains <100 bpm despite facemask ventilation, corrective steps action should be taken to optimize ventilation. If the HR continues to fall below 60 bpm despite facemask ventilation and corrective actions, chest compressions are indicated. 

The development of the HR immediately after birth is closely linked to the oxygenation of the infant. Thus, although SpO_2_ is an important variable to follow during the failed perinatal transition, an ECG is obtained faster, especially in asphyxiated newborns who have poor peripheral perfusion.

Activation of different parts of the autonomic nervous system may influence HR during perinatal transition. While in asphyxia, sympathetic activity may dominate; vagally mediated parasympathetic activation is commonly caused by external stimuli during delivery room interventions. Most clinicians are familiar with this concept through the ever-increasing acknowledgment of the potential harmful effects of unwarranted delivery room airway suctioning. Vagal activation through stimulation of the trigeminal nerve in the face has been postulated as a mechanism behind a commonly observed decrease in HR after the application of a facemask for PPV [25].

Yam et al. [40] and Palme-Kilander and Tunell [41] suggested that neither an HR < 100 bpm in the first minutes of life alone indicates a need for resuscitative measures nor that an HR > 100 bpm is always consistent with cardiorespiratory stability. When comparing HRs in newborns in need of PPV, there has been a change in the recent 30 years. Studies using dry-electrode ECG may report a higher HR; however, some investigators seem to have recently broadened the indications for PPV by questioning the clinical relevance of HR cutoffs of 100 bpm and 60 bpm for initiating PPV and chest compressions, respectively [44].

Despite several advantages, pulse oximetry has some limitations during neonatal resuscitation. Overall, pulse oximetry is a fairly accurate method for delivery room HR measurement and is recommended for HR- and oxygen saturation monitoring during neonatal resuscitation. Although an ECG is routinely used for continuous HR monitoring in the NICU [18], there are limitations to using ECGs to determine HR in the delivery room. Health care workers should be able to recognize these limitations to avoid harm. Pulseless electric activity may occur in very sick, asphyxiated newborns, and in addition to HR, *pulse* should be assessed by other means in such situations.

### Future Directions

As the universal introduction of delayed cord clamping seems to have lowered the HR reference values in the first minutes after birth [5], methods for HR assessment should be more extensively investigated with regard to their reliability at lower HRs. Also, the intervention threshold of 100 bpm (and potentially 60 bpm) in different subgroups of infants (GA, delivery mode) should be re-evaluated. Pulse oximetry slightly underestimates HR compared to an ECG, which cannot explain the considerably higher HR seen in recently published nomograms generated with dry-electrode ECGs. This should be a focus of future studies.

## 5. Conclusions

At birth, a newborn’s heart rate should be evaluated immediately to determine the need for resuscitation and stabilizing measures. As cardiopulmonary transition is affected by the cord management strategy, gestational age, and underlying condition, different heart rate intervention thresholds might be needed in different subgroups of newborn infants.

## Figures and Tables

**Table 1:** Characteristics of seven studies that reported heart rate values the first 300 s of life in mainly non-ventilated term and preterm infants.

Author Year	Method for HR Measurement	*N*	GA, Mean (Range or SD)	BW, Mean (Range or SD)	Results	Cord Clamping	Delivery Mode
Dawson et al., 2010 [9]	PO—sensor right hand and wrist (1–10 min)	Total = 468 Term = 308 Preterm = 160	GA 38 (25–42) wks (all) GA 40 (37–42) wks (term) GA 33 (25–36) wks (preterm)	2970 (625–5135) g	HR rose more slowly in infants born: 1. Preterm; 2. Via caesarian section; 3. To mothers who received anesthetics and narcotics during labor.	ECC No ISSC	NVD/Instrument/CS
Smit et al., 2014 [5]	PO—sensor right hand and wrist (1–10 min)	Leiden = 109 International ref.range = 308	GA 40 (37–42) wks	3575 (482) g	The HR in the Leiden cohort was lower (*p* < 0.05) and increased more slowly in the first 3 min. In the first min of life, tachycardia (HR > 180 bpm) occurred less frequently and bradycardia (HR < 80 bpm) more often (*p* < 0.05)	DCC ISSC	NVD
Mukherjee et al., 2020 [8]	PO—sensor right hand and wrist (30 s–5 min)	DCC-NVD = 170 ECC-NVD = 178 ECC-CS = 101	GA ≥ 34 wks	DCC-NVD; 2540 (2340–2870) g * ECC-NVD: 2600 (2320–2820) g *	The median HR of the DCC-NVD group was significantly lower than in the ECC-NVD group at 1 min and significantly higher at 4 and 5 min. There was no between-group difference according to GA or BW.	DCC ECC	NVD/CS
Badurdeen et al., 2022 [10]	3-lead ECG (1–10 min)	*n* = 295	GA ≥ 35 + 0 wks * GA 39.6 (38.6–40.6) wks *	3.40 [3.08–3.70] kg *	PBCC resulted in similar mean HR compared to ECC in infants receiving resuscitation: Mean HR between 60–120 s after birth was 154 bpm in PBCC vs. 158 bpm in ECC. Percentile chart for HR and SpO2; non-ventilated infants receiving DCC (observational arm group).	DCC	NVD/CS
Bjorland et al., 2020 [11]	NeoBeat (5 s–5 min)	*n* = 898	GA 40 (1) wks	3594 (478) g	During the first 30 s after birth, the HR increased from median (IQR) 122 (98–146) bpm to 168 (145–185) bpm with a maximum of 175 (157–189) bpm 61 s after birth and then slowly decreasing.	DCC ISSC	NVD
Kukka et al., 2023 [13]	NeoBeat (10–180 s)	1. Crying = 115 2. Non-crying breathing = 54	GA ≥ 33 wks 1. GA 39.2 (1.3) 2. GA 39.1 (1.6)	1. 3065.7 (414.8) g 2. 3047.2 (410.5) g	There was no difference in median HR between the groups. Non-crying but breathing infants had higher odds of bradycardia (HR < 100 bpm, adjusted OR 2.64, 95% CI 1.34 to 5.17) and tachycardia (HR > 200 bpm, adjusted OR 2.86, 95% CI 1.50–5.47).	ECC/DCC in both groups	NVD/ Instrument
Van vonderen et al., 2015 ** [12]	PO/ 3-lead ECG	*n* = 48 term/preterm infants	GA 36 (27–41) wks	2848 (1694–3356) g *	First 2 min HRPO was significantly lower than HRECG, 94 (67–144) vs. 150 (91–153) bpm at 60 s. (*p* < 0.001) and 83 (67–145) vs. 158 (119–176) bpm at 120 s. (*p* < 0.001). The largest difference was observed between 60 and 120 s after birth; sixty-four percent of the infants had bradycardia (HR < 100 bpm) according to PO, while only twenty-seven percent had bradycardia according to ECG.	Unknown	NVD/CS

Note: GA (gestational age); BW (birth weight); NVD (normal vaginal delivery); CS (caesarian section); PO (pulse oximetry); ECG (electrocardiogram); NeoBeat (dry-electrode ECG); HRPO (heart rate pulse oximetry); HRECG (heart rate ECG); DCC (delayed cord clamping); ECC (early cord clamping); PBCC (physiologically cord clamping); ISSC (immediate skin-to-skin contact); OR (odds ratio); CI (confidence interval); * GA/BW in median (IQR). ** Study included ventilated infants.

**Table 2:** Heart rate values the first 300 s of life in mainly non-ventilated term and preterm infants.

	Dawson et al., 2010 [9]		Smit et al., 2014 [5]	Mukherjee et al., 2020 [8]		Van Vonderen et al., 2015 ** [12]		Badurdeen et al., 2022 [10]	Bjorland et al., 2020 [11]	Kukka et al., 2023 [13]	
	Term Infants *n* = 308	Preterm Infants *n* = 160	Term Infant, DCC, ISSC *n* = 109	Term/Late Preterm DCC *n* = 170	Term/Late Preterm ECC *n* = 178	Term/Preterm Infant *n* = 53		Term Infants, ≥GA35, DCC *n* = 295	Term Infant DCC, ISSC *n* = 898	Term/Late Preterm Crying	Term/Late Preterm Non-Crying Breathing
Time:	HRPO	HRPO	HRPO	HRPO	HRPO	HRPO	HRECG	HRECG	NeoBeat	NeoBeat	NeoBeat
10 s	-	-	-	-	-	-	-	-	129 (102–154)	157 (118–174)	155 (104–178)
30 s	-	-	-	99 (81–138)	88 (48–120)	-	-	-	168 (146–185)	168 (146–182)	164 (140–173)
60 s	99 (66–132)	96 (72–122)	61 (42–146)	128 (96–153)	140 (127–152)	94 (67–144)	150 (91–153)	171 (156–186)	174 (157–189)	168 (151–182)	162 (149–185)
90 s	127 (94–158)	110 (87–144)	-	-	-	81 (60–109)	148 (83–170)	–	173 (157–187)	169 (154–183)	164 (156–188)
120 s	144 (115–171)	122 (100–144)	85 (67–164)	150 (130–158)	148 (128–159)	83 (67–145)	158 (119–176)	173 (158–187)	171 (156–185)	169 (156–183)	174 (161–191)
180 s	160 (138–180)	142 (122–160)	157 (145–169)	151 (137–158)	146 (129–157)	-	-	172 (158–186)	168 (153–182)	169 (156–182)	170 (161–195)
240 s	163 (145–181)	154 (137–170)	152 (140–163)	154 (140–160)	148 (136–159)	-	-	171 (157–184)	167 (153–181)	-	-
300 s	164 (147–180)	156 (142–171)	150 (140–161)	168 (160–174)	159 (144–168)	-	-	169 (156–182)	167 (152–179)	-	-

Note: HR (bpm); median (IQR), HRPO (heart rate pulse oximetry); HRECG (heart rate ECG, 3-lead); NeoBeat (dry-electrode ECG); DCC (delayed cord clamping); ECC (early cord clamping); ISSC (immediate skin-to-skin contact). ** Study included ventilated infants.

## Data Availability

Not applicable.
