# Correction: Nerdrum Aagaard et al. Significance of Neonatal Heart Rate in the Delivery Room—A Review. *Children* **2023**, *10*, 1551

**DOI:** 10.3390/children11010120

**Published:** 2024-01-18

**Authors:** Ellisiv Nerdrum Aagaard, Anne Lee Solevåg, Ola Didrik Saugstad

**Affiliations:** 1 Division of Pediatric and Adolescent Medicine, Oslo University Hospital Rikshospitalet, 0424 Oslo, Norway; ellaag@ous-hf.no (E.N.A.); a.l.solevag@medisin.uio.no (A.L.S.); 2 Department of Pediatric Research, University of Oslo, 0424 Oslo, Norway; 3 Department of Pediatrics, Robert H Lurie Medical Research Center, Northwestern University, Chicago, IL 60611, USA

## Table Legend

In the original publication [1], there was a mistake in Table 1 as it was published. We referred to the wrong abbreviation, “PBBC”. The corrected “PBCC” appears below.

## Missing Citation

In the original publication [1], “[37] Badurdeen, S.; Brooijmans, E.; Blank, D.A.; Kuypers, K.L.A.M.; Te Pas, A.B.; Roberts, C.; Polglase, G.R.; Hooper, S.B.; Davis, P.G. Heart Rate Changes following Facemask Placement in Infants Born at ≥32 + 0 Weeks of Gestation. *Neonatology*. **2023**, *120*, 624–632.” was not cited. The citation has now been inserted in “3.1. Factors, Including Measurement Method, That Influence HR in Newborn Infants Immediately after Birth”, “3.1.5 Heart Rate in Hypoxemia and Asphyxia” and “3.2. Delivery Room HR as a Prognostic Indicator in Different Subgroups of Newborns”, “3.2.1. The Golden Minute” and these should read:

“Badurdeen et al. [37] investigated the trigemino-cardiac reflex in late preterm and term infants and found that in initially depressed infants, the application of a facemask resulted in an increased not a decreased HR. These findings prompted the authors to speculate that the trigemino-cardiac reflex is suppressed in asphyxiated infants, i.e., those with a low or unstable baseline HR. In contrast, the infants that experienced an HR decrease upon facemask application had a higher baseline HR”. 

“Badurdeen et al. [37] showed that in late preterm and term infants with an anticipated need for resuscitation, respiratory support was initiated at a median (IQR) age of 63 (41–112) s”.

Due to the addition of one reference, the reference index has undergone some changes, but the serial numbers before [37] remain unchanged. We updated the original references [38–44]:

[38] McCarthy, L.K.; Morley, C.J.; Davis, P.G.; Kamlin, C.O.; O’Donnell, C.P. Timing of interventions in the delivery room: Does reality compare with neonatal resuscitation guidelines? *J. Pediatr*. **2013**, *163*, 1553–1557.e1.

[39] Wyckoff, M.H.; Aziz, K.; Escobedo, M.B.; Kapadia, V.S.; Kattwinkel, J.; Perlman, J.M.; Simon, W.M.;Weiner, G.M.; Zaichkin, J.G. Part 13: Neonatal Resuscitation: 2015 American Heart Association Guidelines Update for Cardiopulmonary Resuscitation and Emergency Cardiovascular Care. *Circulation* **2015**, *132*, S543–S560. 

[40] Yam, C.H.; Dawson, J.A.; Schmolzer, G.M.; Morley, C.J.; Davis, P.G. Heart rate changes during resuscitation of newly born infants < 30 weeks gestation: An observational study. *Arch. Dis. Child.-Fetal Neonatal Ed*. **2011**, *96*, F102–F107. 

[41] Palme-Kilander, C.; Tunell, R. Pulmonary gas exchange during facemask ventilation immediately after birth. *Arch. Dis. Child*. **1993**, *68*, 11–16.

[42] Kapadia, V.; Oei, J.L.; Finer, N.; Rich, W.; Rabi, Y.; Wright, I.M.; Rook, D.; Vermeulen, M.J.; Tarnow-Mordi, W.O.; Smyth, J.P.; et al. Outcomes of delivery room resuscitation of bradycardic preterm infants: A retrospective cohort study of randomised trials of high vs. low initial oxygen concentration and an individual patient data analysis. *Resuscitation* **2021**, *167*, 209–217. 

[43] Oei, J.L.; Finer, N.N.; Saugstad, O.D.; Wright, I.M.; Rabi, Y.; Tarnow-Mordi, W.; Rich, W.; Kapadia, V.; Rook, D.; Smyth, J.P.; et al. Outcomes of oxygen saturation targeting during delivery room stabilisation of preterm infants. *Arch. Dis. Child.-Fetal Neonatal Ed*. **2018**, *103*, F446–F454. 

[44] Saugstad, O.D.; Kirpalani, H. Searching for evidence in neonatology. *Acta Paediatr*. **2023**, *112*, 1648–1652. 

## Text Correction

There was an error in the original publication [1], Section 3.1.1, where there are two mistakes: “At one minute of age, 61% of healthy term infants had an HR < 100 bpm, decreasing to 21% and 7% at 2 and 3 min of age, respectively (Table 1)”, and “1% of healthy term infants had an HR < 100 bpm [7]”.

A correction has been made to “3.1.1 First Reports of Normal HR Immediately after Birth—Pulse Oximetry”.

“At one minute of age, 61% of healthy term infants had an HR < 100 bpm, decreasing to 21% and 7% at 2 and 3 min of age, respectively, and 1% of healthy term infants had an HR < 100 bpm [9]”.

The authors state that the scientific conclusions are unaffected. This correction was approved by the Academic Editor. The original publication has also been updated.
